# The Toxicological Intersection between Allergen and Toxin: A Structural Comparison of the Cat Dander Allergenic Protein Fel d1 and the Slow Loris Brachial Gland Secretion Protein

**DOI:** 10.3390/toxins12020086

**Published:** 2020-01-28

**Authors:** Holger Scheib, K. Anne-Isola Nekaris, Johanna Rode-Margono, Lotten Ragnarsson, Kate Baumann, James S. Dobson, Wirdateti Wirdateti, Amanda Nouwens, Vincent Nijman, Paolo Martelli, Rui Ma, Richard J. Lewis, Hang Fai Kwok, Bryan Grieg Fry

**Affiliations:** 1 Venom Evolution Lab, School of Biological Sciences, University of Queensland, St Lucia, Qld 4072, Australia; holger@moltalk.org (H.S.); Kate.baumann@outlook.com (K.B.); james.dobson@uqconnect.edu.au (J.S.D.); 2 Nocturnal Primate Research Group, Department of Social Sciences, Oxford Brookes University, Oxford OX3 0BP, UK; anekaris@brookes.ac.uk (K.A.-I.N.); johanna.margono@gmail.com (J.R.-M.); vnijman@brookes.ac.uk (V.N.); 3 Centre for Functional Genomics, Department of Health and Life Sciences, Oxford Brookes University, Oxford OX3 0BP, UK; 4 The North of England Zoological Society / Chester Zoo, Chester CH2 1LH, UK; 5 Institute for Molecular Biosciences, University of Queensland, St Lucia QLD 4072, Australia; l.ragnarsson@imb.uq.edu.au (L.R.);; 6 Research Center for Biology-LIPI, Jakarta-Bogor, Cibinong 16911, Indonesia; wirdateti@lipi.go.id; 7 School of Chemistry and Molecular Biosciences, University of Queensland, St Lucia, Qld 4072, Australia; a.nouwens@uq.edu; 8 Veterinary Department, Ocean Park, Hong Kong; paolo.martelli@oceanpark.com.hk; 9 Institute of Translational Medicine, Faculty of Health Sciences, University of Macau, Avenida de Universidade, Taipa, Macau SAR; yb67598@um.edu.mo

**Keywords:** allergen, dander, evolution, primate, venom

## Abstract

Slow lorises are enigmatic animal that represent the only venomous primate lineage. Their defensive secretions have received little attention. In this study we determined the full length sequence of the protein secreted by their unique brachial glands. The full length sequences displayed homology to the main allergenic protein present in cat dander. We thus compared the molecular features of the slow loris brachial gland protein and the cat dander allergen protein, showing remarkable similarities between them. Thus we postulate that allergenic proteins play a role in the slow loris defensive arsenal. These results shed light on these neglected, novel animals.

## 1. Introduction

The origin and evolution of animal defensive and predatory toxins has been the subject of intense research [1], but that of mammals, possibly because of its infrequent occurrence, has been somewhat neglected. Employing venoms has evolved several times independently in mammals, including in solenodons, platypus, vampire bats, water shrews, and a group of primates, the slow lorises [1,2,3,4,5,6,7]. A slow loris when threatened, raises its arms over the head, bringing sebaceous glands located in the brachial region of the upper arm close to its mouth, the fluid from these brachial glands is mixed with saliva. The fluid is delivered in a bite with procumbent anterior incisors known as the toothcomb, which is believed to act as an effective venom delivery system [8]. Although ecological functions may vary, the toxin is certainly used for intraspecific competition [9]; use in defence against predators is possible but this requires future to determine. Bitten animals suffer from chronically non-healing wounds, leading to necrosis, septicaemia, lung oedema, and cellulitis [10,11]. Envenomation in humans may be followed by paraesthesia, haematuria, dyspnoea, extreme pain, and infection, and in the worst cases can lead to severe or even near-fatal anaphylactic shock [12,13,14]. Healing time may reach several months [13].

Despite this interest in the field, the nature of the secretions produced by the brachial glands of slow lorises has been studied only to a limited detail from captive animals. The secretions produce a characteristic odour that has been shown to be due to a complex array of over two hundred aromatic molecules [15]. While the aromatic compounds have been well-characterised, the protein secretions are known from only small N-terminal fragments details [15]. BLAST searches [16] revealed both chains to belong to the same allergenic protein family as does the *Felis catus* dander allergen Fel d1. Fel d1 is a 35 kDa secretoglobin protein produced by sebaceous, salivary, perianal, and lachrymal glands as well as squamous epithelial cells, and is distributed by *F. catus* over its fur during grooming and licking [17,18,19,20]. Structurally *F. catus* dander allergen Fel d1 is a heterodimer formed by three inter-chain disulphide bonds. Chain One is 70 residues while chain two is 90 or 92 residues long [21,22]. It has been shown that the two Chains One and Two are encoded for by different genes [23]. Two copies of this heterodimer non-covalently associate to form a larger homodimer consisting of Chains A and B. Several attempts have been made to determine the 3D-structure of *F. catus* dander allergen Fel d1 [24,25]. In principle, two ways exist to form a heterodimer from Chain One and Two: Chain One followed by Chain Two or vice versa. When recombinant proteins were investigated both chain arrangements yielded very similar biochemical, immunological, and structural results, even when compared to their natural counterpart [26]. Only Chain 1 + 2 arrangement resulted in forming homodimers [24,25]. Thus, Chain A and B each consists of a Chain 1 + 2 heterodimers.

The limited sequence information is insufficient to reconstruct the molecular evolutionary history of the slow loris brachial gland secreted proteins and therefore their relationship to *F. catus* dander allergen Fel d1 and other proteins within the broader allergen family. In this study, we therefore elucidate the full-length sequence of both chains of the Javan slow lorises’ (*Nycticebus javanicus*) brachial gland secreted proteins, with samples from wild animals. We discuss our findings in the light of available information from the cat *F. catus* dander allergens possibly leading to insights not only on the molecular evolution of mammalian allergens but also gaining a greater understanding how to treat humans when envenomated by slow lorises.

## 2. Results and Discussion

Through a combination of MS/MS sequencing and DNA sequencing, we obtained the first full-length sequences of brachial gland protein of *N. javanicus*, with the full nucleotide sequence of Chain-One determined as (tgtcccgccgtagaaaaacacgctaacctcttcctgaagggaaccactgatgaatttctcaattatgcgaaaaatttcgtaaaatcctctgcagtattggaaaatgctaagcaactgaagatgtgttccgacaataaactgacagaagaggataaggataatgtccagtctgggctggacaaaatatactcaagcaatttttgt) while Chain Two was sequenced using protein sequencing (Table 1).

We then compared them structurally to the homologous *F. catus* dander Fel d1 protein. To date, eight cat dander allergens have been found. They were named Fel d1 to Fel d8 [27,28]. Fel d1 belongs to the secretoglobulin family, Fel d2 is a serumalbumin, Fel d3 a cystatin, Fel d4 and 7 are lipocalins, Fel d5 and 6 are Ig antibodies of type A and M, respectively. Further, Fel d8 is a latherin. Despite Fel d1 Fel d4 is the second most prominent cat dander allergen with 63% of people allergic to cats have formed antibodies against [29]. Fel d1 was discovered in 1973 and is the most prominent of these allergens [30]. It accounts for 96% of cat allergies [31]. It is found in every house and public place, regardless of the presence of cats. Fel d1 has spread globally and was detected even in regions where cats most likely have never lived, i.e., the Greenland inland ice shelf. This has been associated with particle sizes of Fel d1 that can reach less than 4 cm in diameter and thus are susceptible to eolian distribution [32]. Fel d1 is produced by every cat but the amount produced varies greatly with race, age, and sex. Adult cats produce more Fel d1 than kittens and male cats more than females. Neutered cats probably produce similar amounts of Fel d1 than females [33]. It has been postulated that Siberian and Balinesian cats produced less Fel d1 than most other races. However, the body of data is still sparse and the topic remains unclear [27]. Any similarity of brachial gland protein of *N. javanicus* and cat allergen Fel d1 hints towards a better understanding of the mechanism of action of brachial gland protein of *N. javanicus* as well as possible treatments to slow loris bites in humans.

**Figure 1:**
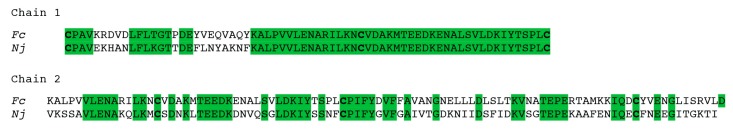
Sequence alignment of *Felis catus* (Fc) dander allergen Fel d1 and *Nycticebus javanicus* (Nj) brachial gland secretion protein. Identical residues are shaded in grey.

Sequence alignment of target to templates revealed that the cysteine residues are conserved indicating that disulfide bridges are conserved as well (Figure 1). The *N. javanicus* brachial gland protein chains are shorter than the *F. catus* allergen structures as Chain One is missing the first three N-terminal residues M0, E1, and I2 as well as the C-terminally located V71 to T76. Chain One of the *N. javanicus* brachial secretion protein contains 36 out of 68 identical residues corresponding to 52.9% sequence similarity. In Chain Two, 25 out of 70 residues (35.7%) are identical.

**Figure 2:**
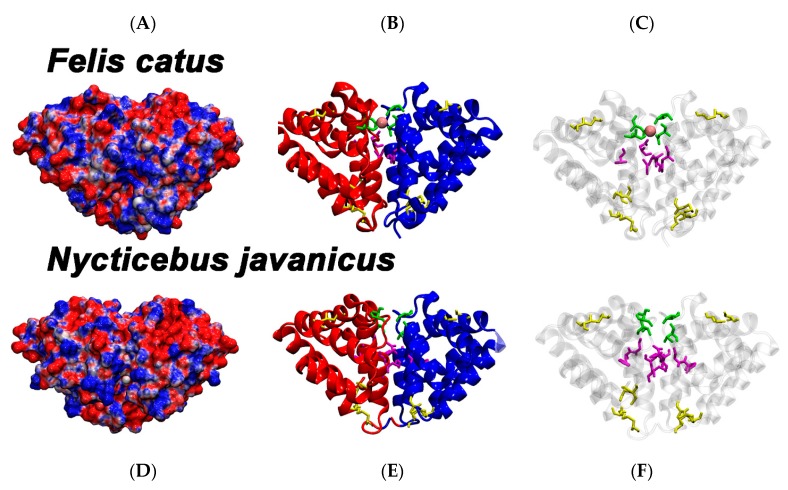
Molecular modelling comparison of *Felis catus* dander allergen Fel d1 (top, **A**–**C**) and *Nycticebus javanicus* brachial gland secretion protein (bottom, **D**–**F**). A and D show the electrostatic surface of both proteins. Positive charges are depicted in blue, negative charges in red. B and E show cartoon representations of the same proteins. Domain A is shown in red, Domain B in blue. C and F are identical to B and E, but with transparent tertiary structure. B, C, E, and F: disulfide bridges are highlighted in yellow. B and C: N89 and I125 of Domain A and N89 and D130 of Domain B form the Ca^2+^ binding site at the domain interface in *F. catus*. These four residues are shown in green with the Ca^2+^ ion in pink. Six amino acids forming the hydrophobic core at the interface of Domains A and B are coloured in magenta (F85, G131, and L132 in both Domains A and B, respectively) E and F: possible Ca^2+^ binding in *N. javanicus* involves T81 and T117 of Domain A and T81 and D123 of Domain B. The hydrophobic core may consist of G77, D123, and I124 in both Domains A and B. D123 as a charged amino acid is expected to affect the interface in *N. javanicus*.

In order to examine the structural evolution of the *N. javanicus* brachial secretion protein, the *F. catus* dander allergen Fel d1 structure template 2EJN was first downloaded from the protein databank (https://www.rcsb.org) [34] consisting of a heterodimeric homodimer (two non-covalently linked copies of a covalently linked heterodimer) (Figure 2). The *F. catus* dander allergen Fel d1 heterodimer consists of two chains (A and B) linked to each other by three disulfide bonds within each heterodimer. All three disulfide bonds link together in the format of Chain A cysteine^1^ – Chain B cysteine^3^; Chain A cysteine^2^ – Chain B cysteine^3^; and Chain A cystine^3^ – Chain B cysteine^1^. Each domain consists of eight α-helices, for each to Chain One and Two, respectively. A Ca^2+^-binding site at the domain interface consists of I125 of Domain A and D130 of Domain B as well as N89 from both Domain A and B and three water molecules [25]. The same structure contains a hydrophobic core consisting of six residues: F85 (Domain A and B), G131 (Domain A and B), and L132 (Domain A and B) that are surrounded by hydrogen bond forming amino acids. This core forms the central structural element of the dimerization site [25]. More remote Ca^2+^-binding Sites Two and Three involved D46 and M49 in each of the domains [25]. In addition, there is a glycosylation site at position N103, at a surface loop connecting helices H6 and H7 [21,35]. Of particular importance for interpreting the diversification of the *N. javanicus* brachial gland protein, cavities were identified at the same region in each domain of *F. catus* dander allergen Fel d1 2EJN [25]. The volume of these cavities differed significantly in size (350 Å^3^ in Domain A versus 730 Å^3^ in Domain B) due to the differing conformations of the eleven residue long stretch from E121 to G131 in both domains. Kaiser et al. hypothesised that these cavities may host progesterone [24,25]. This result would be in accordance to findings of uteroglobin and other orthologues such as CC16 (Clara cell secretory protein) and ABP (androgen-binding protein) that bind progesterone, PCB-derivatives (PCB: polychlorinated biphenyl) and androgen [36,37,38,39,40].

When modelled, as the *N. javanicus* brachial gland protein chains are shorter than the *F. catus* allergen chains, the structures shown for both proteins appear slightly different in shape, despite being superimposed and rotated the same way, but overall the structures are highly similar (Figure 2), which is reflective not only of shared molecular evolutionary history but suggestive of similar functionality. In the *N. javanicus* brachial secretion protein the critical residue for PLA_2_ inhibition D44 (homologous residue D46 in Fel d1) is held in position by two salt bridges from K40 and K52. In *F. catus* dander allergen Fel d1 the homologous stabilizing residues are K42 and K54.

Comparative examination of the Ca^2+^-binding site revealed that in the *F. catus* dander allergen Fel d1, the Ca^2+^-binding site 1 is located in a prominent position at the contact area between Domains A and B. Four residues form Ca^2+^-binding site 1: the side chain carbonyl atom of N89 and backbone carbonyl atom of I125 of Domain A as well as the side chain carbonyl atoms of N89 and D130 of Domain B. Since the *N. javanicus* brachial secretion protein was modelled without Ca^2+^-ions present, the attractive force of Ca^2+^ is missing, affecting the side-chain placement of T81(A), T81(B), and D123(B). Like I125 in *F. catus* dander allergen Fel d1, the respective T117 (A) orients its backbone carbonyl toward Ca^2+^-binding Site One. Despite these differences in side chain orientation, we postulate that Ca^2+^-binding Site One is also present in the dimerization area of the *N. javanicus* brachial secretion protein.

Comparative examination of the hydrophobic core revealed that the *F. catus* dander allergen Fel d1 a hydrophobic core made of six residues, namely F85, G131, and L132 of both Domains A and B [25]. In the *N. javanicus* brachial secretion protein this interdomain region is not all hydrophobic: G77 (A and B), D123 (A and B), I124 (A and B). Moreover, it is differently shaped. Where in *F. catus* dander allergen Fel d1 the large phenylalanine side chain reaches deep into the cavity, in the *N. javanicus* brachial secretion protein there is a glycine. By contrast, the position homologous to G131 of *F. catus* dander allergen Fel d1 is in the *N. javanicus* brachial secretion protein occupied by D123. We postulate that for the *N. javanicus* brachial secretion protein the hydrophobic core, therefore, differs from that of the allergen of *F. catus*.

Comparative examination of the dimerization site revealed that the two heterodimeric Domains A and B of *F. catus* dander allergen Fel d1 and that of the *N. javanicus* brachial secretion protein form a homodimer. The interface between the two domains consists of 21 amino acids of Domain A (S29, A31, Y73, G74, G77, T81, D83, I86, F90, T117, G118, K119, T120, D123, I124, I126, G127, A130, I131, I136, I137) and 20 residues of Domain B (same residues, except I137). Accessible surface area was calculated for both domains separately and the homodimer (Table 2). As expected, the number of solvent exposed surface atoms decreases upon dimerization. This is reflected in a 14% decrease of total accessible area as compared to the two separate heterodimeric Domains A and B.

Comparative examination of the glycosylation site revealed that in *F. catus* dander allergen Fel d1 the glycosylation site N103 lies in the surface loop between helices H6 and H7. The corresponding residue in the *N. javanicus* brachial secretion protein is S95. Serine can be O-glycosylated and therefore glycosylation may occur at the same structural position as in *F. catus* dander allergen Fel d1 structure 2EJN.

Kaiser et al. postulated immunoglobulin E (IgE)-binding to solvent exposed residues [24]. IgE is an antibody that is known to play a crucial role in type I hypersensitivity. Type I hypersensitivity has been linked to allergic asthma, sinusitis, allergic rhinitis, food allergies, chronic urticaria, and atopic dermatitis. It is involved in allergen response, e.g. bee stings or anaphylactic drugs [42]. The apparent similarity of residues in *F. catus* dander allergen Fel d1, and the *N. javanicus* brachial secretion protein hints toward a similar function. *F. catus* dander allergen Fel d1 was identified several decades ago and is responsible for the immunoglobulin IgE-response in 90–95% of patients who are allergic to *F. catus* [31,43]. The body of work carried out in this study revealed that not only *F. catus* dander allergen Fel d1 may be able to trigger an immune response. A number of proteins exist in evolutionary distant animals that potentially can act similar to the major *F. catus* allergen. Of particular note is the molecular similarity between the *N. javanicus* brachial secretion protein and *F. catus* dander allergen Fel d1 which suggests a functional similarity that is consistent with known reactions to *Nycticebus* bites. We postulate that approaches to treat cat allergy, such as desensitization, may work for researchers and caretakers of slow lorises as well [44,45,46]. Furthermore, understanding the causes of allergic reactions up to anaphylactic shock greatly enhances chances for successful treatment of slow loris’ bites.

Future work should investigate the potential role of immunological feedback loops that are overstimulated and may explain the festering, non-healing wounds characteristic of slow loris bites. Treatment with cat antiallergens may be a starting point.

## 3. Materials and Methods

### 3.1. Brachial Gland Secretion

We obtained brachial gland secretions of *Nycticebus javanicus* from wild slow lorises studied at the ecological research station in Cipaganti, Garut Regency, West Java, Indonesia (S7°6’6–7°7’0& E 107°46’0–107°46’5). We collected samples used here from April 2012 to June 2014, during which time we captured the animals for health checks associated with a radio-tracking project [47]. After capture, we manually held the non-anaesthetized animals and gently used a sterile swab to collect the brachial secretion (supplementary video 1). Each swab was then contained, sealed with parafilm, and frozen at −80 °C. Samples were exported from Indonesia to Australia in August 2016 with approval from the Indonesian Ministry of Forestry, Indonesia’s CITES Management Authority, and Australia’s Department of Environment. The work was conducted under the RISTEK research permits 039/SIP/FRP/SM/11/2012, 11/TKPIPA/FRP/SM/11/2013, and 163/SPP/RPB/WU/5/2014. The Animal Ethics Subcommittee of Oxford Brookes University’s University Research Ethics Committee approved this research.

### 3.2. MS/MS 

Protein was first desalted on C8 column, and resuspended in 100 mM ammonium bicarbonate. Protein was reduced with 10 mM DTT, 95 °C, 15 min, cooled to room temperature, and alkylated with iodoacetamide (25 mM final concentration) for 30 min, RT in the dark. Additional DTT (1ul, 100 uM) was added to quench excess iodoacetamide. The sample was split into aliquots, and digestion was performed overnight at 37 °C with either trypsin, chymotrypsin, AspN, GluC or LysC. Samples were ziptipped (C18, 0.6 ul resin, MerckMillipore) before analysis by mass spectrometry.

### 3.3. LC-MS/MS Analysis

Peptides were separated using reversed-phase chromatography on a Shimadzu Prominence nanoLC system. Using a flow rate of 30 µL/min, samples were desalted on an Agilent C18 trap (0.3 × 5 mm, 5 µm) for 3 min, followed by separation on a Vydac Everest C18 (300 A, 5 µm, 150 mm × 150 µm) column at a flow rate of 1 µL/min. A gradient of 10–60% buffer B over 30 min where buffer A = 1 % ACN / 0.1% FA and buffer B = 80% ACN / 0.1% FA was used to separate peptides. Eluted peptides were directly analyzed on a TripleTof 5600 instrument (ABSciex) using a Nanospray III interface. Gas and voltage settings were adjusted as required. MS TOF scan across m/z 350–1800 was performed for 0.5 sec followed by information dependent acquisition of up to 20 peptides across m/z 40–1800 (0.05 sec per spectra). 

LC-MS/MS data was de novo sequenced using PeaksStudio software, with enzyme specified based on sample, mass tolerance of 20 ppm, fragment mass tolerance of 0.1 Da, and variable modifications including oxidation of methionine, and carbamidomethylation set as fixed modification. Resulting de novo sequences were manually curated and validated, with sequences BLAST searched to determine other proteins with similarity.

### 3.4. mRNA 

The 3’ rapid amplification of cDNA ends (RACE) first strand cDNA was synthesized from 34 ng total RNA using the FirstChoice RLM-RACE kit (Ambion), following the manufacturer’s instructions. The resulting cDNA was used as template in a polymerase chain reaction (PCR) using a forward primer (F) designed from the partial α-chain venom peptide sequence and a reverse 3’RACE outer primer supplied in the FirstChoice RLM-RACE kit (Ambion). The known peptide sequence was reverse-translated into a nucleotide sequence for primer design. Primer sequences were F=5’-GGTGGAAAAACATGCGAAC-3’ and 3’RACE outer primer = 5’-GCGAGCACAGAATTAATACGACT-3’. The PCR reaction was performed using FastStart Taq DNA polymerase (Roche) with 3’RACE-cDNA as template under the following cycling conditions: 95 °C for 4 min, followed by 40 cycles of 95 °C for 30 s, 58 °C for 30 s, and 72 °C for 1 min and a final elongation step at 72 °C for 7 min. PCR products were analyzed and purified after separation on a 1% agarose gel using a QIAquick Gel Extraction kit (Qiagen) and cloned into vector pCR2.1 (Invitrogen) for sequencing by the Australian Genome Research Facility using an M13 forward primer.

### 3.5. Modeling

In order to structurally modelling of Chain 1 + 2 heterodimers and homodimers of Chain A and B, the target sequence was aligned to the template sequence of the major *F. catus* allergen Fel d1 in Swiss PDB Viewer [48]. The major *F. catus* allergen Fel d1 template structures 1ZKR and 2EJN [24,25] were downloaded from the PDB (www.rcsb.org) [34]. It was used as the template for all molecules in this study. The target sequences A and B were aligned to the template and target to template sequence alignments were exported in gapped FASTA-format from SPDBV [48]. The alignment was read in Chimera (University of San Francisco), version 1.8 [49]. Models were created using MODELLER [50] through UCSF Chimera as a Graphical User Interface. Due to the overall high sequence identity between target and template the series of models obtained from MODELLER were very similar. Therefore, the first model with the lowest overall energy was chosen. The model structures were generated in SPDBV [48] and side chains were cleaned up by following energy minimization of 20 and two times 100 steps of steepest descent minimization as implemented in SPDBV. Interface residues were identified by calculating contact surfaces of neighbouring domains in the homodimer using InterProSurf [51]. Hydrophobic patches were identified by calculating the respective surfaces in SPDBV. Surface charges were calculated using the APBS-algorithm [52] as implemented in VMD [53]. The calculated potential-maps were loaded to the protein under inspection. Surfaces were visualized applying the “surf” option and colouring method “volume” in VMD. The range for positive (blue) to negative charges (red) was set to −20.00 and 20.00, respectively.

## Figures and Tables

**Table 1:** Protein sequencing results for Chain 2.

Enzyme Digest	Initial de novo Sequence Returned by Peaks Studio	ALC (%)	m/z	z	Final Correct Sequence
Trypsin	N(+42.01) TLFYGVFGALVTGDK	96	872.4631	2	CPIFYGVFGAIVTGDK
Trypsin	NLLDSFLDKVSGTEPEK	96	631.3182	2	NIIDSFIDKVSGTEPEK
Trypsin	T(+42.01)TC(+71.06)KLQM(+15.99)AAFNEEGLTGK	92	762.8611	2	IQECFNEEGITGK
Trypsin	LTEEDQDNVQSGLDK	97	564.2603	3	LTEEDKDNVQSGLDK
Trypsin	Edman degradation				VKSSAVLENAK
Trypsin	Edman degradation				IYSSNFCPIFYGVFGAIVTGDK
AspN	DNKLTEEDKN(+.98)NVQSGL	94	902.9345	2	DNKLTEEDKDNVQSGL
LysC	LTEEDKDNVQSGLDK	98	845.91	2	LTEEDKDNVQSGLDK
LysC	AAFENLKEC (+57.02) FNEEGLTGK	96	1028.9913	2	AAFENIQECFNEEGITGK

**Table 2:** Accessible surface area of isolated heterodimer Domains A and B and the homodimer. Surface areas were calculated using InterProSurf [41].

Chain	Polar Area/Energy	Apolar Area/Energy	Total Area/Energy	Surface Atoms	Buried Atoms
Domain A	2602.53	4497.99	7100.53	609	439
Domain B	2602.62	4497.99	7100.61	609	439
Homodimer	4730.58	7466.48	12197.06	1170	926
